# Personality and psychophysiological self-regulation influence individual efficacy of neurofeedback in tension-type headache

**DOI:** 10.1192/j.eurpsy.2021.1314

**Published:** 2021-08-13

**Authors:** G. Arina, O. Dobrushina, E. Shvetsova, G. Meshkov, S. Martunov, E. Fyodorova

**Affiliations:** 1 Psychology, Lomonosov Moscow State University, Moscow, Russian Federation; 2 Neurology, International Institute of Psychosomatic Health, Moscow, Russian Federation; 3 Data Science, International Institute of Psychosomatic Health, Moscow, Russian Federation; 4 Psychology, International Institute of Psychosomatic Health, Moscow, Russian Federation

**Keywords:** tension-type headache, Neurofeedback, Personality, psychophysiological self-regulation

## Abstract

**Introduction:**

Due to limited efficacy and side effects of pharmacological therapy in tension-type headache (TTH), alternative approaches are feasible. Neurofeedback is a noninvasive neuromodulation technique increasingly used in practice, but, however, there is limited research on its efficacy.

**Objectives:**

To evaluate the efficacy of neurofeedback in TTH and to reveal the factors moderating treatment effects.

**Methods:**

We analyzed the data from a pilot phase of an ongoing single case design cross-over sham-controlled study. Four females with TTH underwent 10 sessions of neurofeedback and 10 sessions of sham-neurofeedback in a randomized order. Participants filled a detailed headache diary 3 weeks before, during and 3 weeks after the treatment. At enrollment, we evaluated the personality factors with the MMPI, and performed a specially developed test on psychophysiological regulation of breath.

**Results:**

Significant reduction of headache frequency and intensity was observed in 2 of 4 participants (responders). The responders were characterized by normal MMPI profile and, the same time, by lower baseline abilities for psychophysiological self-regulation. The non-responders had high MMPI profile (accentuation) and also higher abilities for psychophysiological self-regulation.

**Conclusions:**

On the base of preliminary data, we suggest that neurofeedback may be feasible in TTH patients with lowered abilities for in psychophysiological self-regulation. Accentuation of personality traits may interfere with the efficacy of neurofeedback.

